# Using Biotinylated Iron-Responsive Element to Analyze the Activity of Iron Regulatory Proteins

**DOI:** 10.3390/ijms25094852

**Published:** 2024-04-29

**Authors:** De-Liang Zhang, Hayden Ollivierre, Tracey A. Rouault

**Affiliations:** *Eunice Kennedy Shriver* National Institute of Child Health and Human Development, National Institutes of Health, Bethesda, MD 20892, USA; deliang.zhang@nih.gov (D.-L.Z.); hayden.ollivierre@nih.gov (H.O.)

**Keywords:** iron metabolism, iron regulatory protein, iron regulatory element, IRE-binding activity, IRP1, IRP2

## Abstract

Iron regulatory proteins (IRP1 and IRP2) are the master regulators of mammalian iron homeostasis. They bind to the iron-responsive elements (IREs) of the transcripts of iron-related genes to regulate their expression, thereby maintaining cellular iron availability. The primary method to measure the IRE-binding activity of IRPs is the electrophoresis mobility shift assay (EMSA). This method is particularly useful for evaluating IRP1 activity, since IRP1 is a bifunctional enzyme and its protein levels remain similar during conversion between the IRE-binding protein and cytosolic aconitase forms. Here, we exploited a method of using a biotinylated-IRE probe to separate IRE-binding IRPs followed by immunoblotting to analyze the IRE-binding activity. This method allows for the successful measurement of IRP activity in cultured cells and mouse tissues under various iron conditions. By separating IRE-binding IRPs from the rest of the lysates, this method increases the specificity of IRP antibodies and verifies whether a band represents an IRP, thereby revealing some previously unrecognized information about IRPs. With this method, we showed that the S711-phosphorylated IRP1 was found only in the IRE-binding form in PMA-treated Hep3B cells. Second, we found a truncated IRE-binding IRP2 isoform that is generated by proteolytic cleavage on sites in the 73aa insert region of the IRP2 protein. Third, we found that higher levels of SDS, compared to 1–2% SDS in regular loading buffer, could dramatically increase the band intensity of IRPs in immunoblots, especially in HL-60 cells. Fourth, we found that the addition of SDS or LDS to cell lysates activated protein degradation at 37 °C or room temperature, especially in HL-60 cell lysates. As this method is more practical, sensitive, and cost-effective, we believe that its application will enhance future research on iron regulation and metabolism.

## 1. Introduction

Iron is an essential trace element for all living organisms as it plays a crucial role in various physiological processes, including oxygen transport, mitochondrial respiration, nucleotide synthesis, and others. However, as excess iron can promote oxidative stress and damage cells and organs, iron levels need to be tightly controlled in vivo. Iron regulatory proteins (IRP1 and IRP2) regulate cellular iron availability by binding to the iron-responsive elements (IREs) of mRNAs of iron-related genes and regulating their protein expression in response to cellular iron levels [1]. The IRE is a stem-loop secondary mRNA structure that contains a conserved six-nucleotide loop (“CAGUGN”, in which N could be C, A, or U but not G), followed by a stem and a conserved C-bulge five nucleotides upstream. Under iron deficiency conditions, binding of IRPs to IREs inhibits the translation of mRNAs with 5′-IRE stem loops such as ferritins and ferroportin (FPN), whereas it stabilizes the mRNAs that contain 3′-IREs such as the transferrin receptor (TfR1) and divalent metal transporter (DMT1). Thus, IRP binding inhibits iron storage and export and increases iron absorption to maintain cellular iron availability [2,3,4].

IRP1 and IRP2 share 60% homology but are regulated differently. IRP1 has a cubane [4Fe-4S] cluster that serves as an iron sensor, which enables IRP1 to work as cytosolic aconitase under iron-replete conditions. Under iron depletion conditions, IRP1 loses its iron–sulfur cluster and converts to an IRE-binding protein. By contrast, IRP2 does not have an iron–sulfur cluster, but it undergoes degradation under iron-replete conditions via the ubiquitination and proteasomal pathway mediated by the F-box and leucine-rich repeat protein 5 (FBXL5), which senses iron and oxygen levels and targets IRP2 for degradation in complex with the SKP1-CUL1-ubiquitin ligase [5,6,7]. The activity of IRPs has generally been measured by the electrophoresis mobility shift assay (EMSA), in which IRPs form a complex with a radioisotope ^32^P-labeled IRE probe and shift slowly to separate from the free probes in non-denaturing electrophoresis [8]. However, the utilization of radioactive isotopes prevents many researchers from performing this assay. In addition, the probe has to be prepared every month due to the short half-life of the ^32^P isotope. The biotinylated-IRE probe was previously used as a method for the original affinity purification of IRE-binding proteins [9]. Recently, the biotinylated-IRE probe has also been used for affinity purification of IRE-binding proteins and for analyzing their activities in a few research articles [10,11,12,13]. However, the lack of technical optimization and details has prevented this method from becoming widely used in iron metabolism research.

Here, we analyzed the ratio and incubation order of the probe, beads, and cell lysates and optimized the protocol to measure IRP activity using the biotinylated IRE. Using this method, we successfully analyzed the IRE-binding activity of cultured cells and mouse tissues treated with various iron conditions. Unlike the regular radiolabeled EMSA method, this method does not require a radioactive isotope, and the probe has a longer probe shelf life, making it more practical and cost-effective. Moreover, because this method separates IRE-bound IRPs from the rest of the cell lysates, it dramatically enhances the specificity of antibodies and has revealed some previously unrecognized information.

## 2. Results

### 2.1. Optimizing the Method of Using Biotinylated IRE to Analyze IRE-Binding Activity

**Figure 1:**
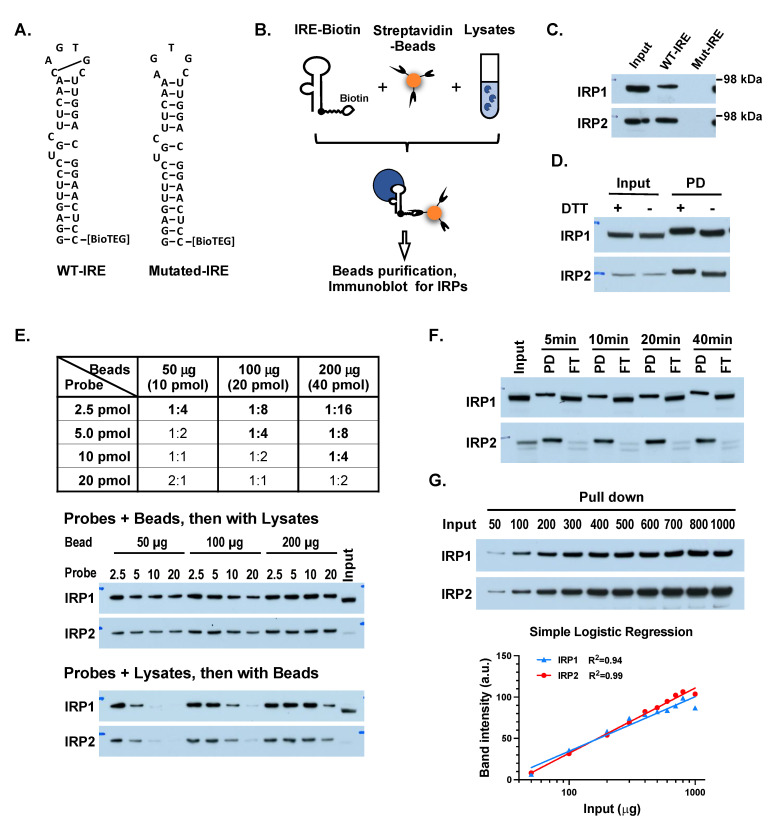
Optimization of assay using biotinylated-IRE probe to isolate and measure IRE-binding protein. (**A**) Wild-type (WT) and mutant IRE with 3′-biotinylation. (**B**) Scheme to isolate and measure IRPs from cell lysates. (**C**) WT but not mutant IRE isolated and detected IRE-binding IRPs from Hep3B cell lysates. (**D**) Addition of 50 mM DTT in the loading buffer slowed the mobility of isolated IRPs, suggesting disulfide bonds were present in IRP proteins. PD, biotinylated-IRE pull-down fraction. (**E**) Test the ratio between beads and IRE probe, and the incubation order between IRE probe, beads and cell lysate to reveal the most sensitive combination on measuring IRP activity. Bracket, the binding affinity of the beads on single-strand-oligonucleotide. Results indicated that incubating biotinylated-IRE probe with Streptavidin-beads first in a ratio of 1:4 provided the best sensitivity. (**F**) Incubation of probe-bead complexes with lysates for as short as 5 min depleted the IRE-binding forms of IRPs from the lysates. Input: 25 μg Hep3B lysates for panel (**C**,**D**,**F**). (**G**) 5 pmol probes/100 μg beads could measure IRE-binding activity semi-quantitively in a wide range of Hep3B lysates. Bottom, Simple Logistic Regression of the band intensities vs. input lysate.

To check the feasibility of using the biotinylated IRE to isolate IRPs, followed by Western blot to analyze the IRE-binding activity, 10 pmol biotinylated wild-type (wt)-IRE or mutated-IRE (mut-IRE) was incubated with 200 µg DynaBead M280 beads for 20 min, followed by washing and then incubating with 100 µg Hep3B cell lysates for 20 min (Figure 1A,B). The bead-IRE-IRP complexes were then washed, resuspended in 100 µL 1x LDS loading buffer, and denatured at 85 °C for 5 min, and then 25 µL (equivalent to 25 µg of the input) along with 25 µg of the input in 1x LDS loading buffer was loaded to Bis-Tris gel for electrophoresis and immunoblotting to measure IRPs. Results (Figure 1C) showed that the wt-IRE probe successfully isolated both IRP1 and IRP2, whereas mut-IRE could not. While the IRP2 band intensities of IRE-pull-down (PD) samples were similar to the input, the IRP1 band intensity of PD samples was weaker than that of the input, which is consistent with the fact that IRP1 is a bifunctional enzyme, and only a portion of IRP1 is in the IRE-binding form [14]. Of note, the IRP1 and IRP2 bands in the IRE-PD samples shifted slightly slower in comparison to the input lane (Figure 1C), which is likely due to disulfide bonds, since the removal of DTT from the loading buffer shifted the IRP1 and IRP2 bands in PD samples to match the position found in the input (Figure 1D).

To optimize the method, we tested: (1) Different ratios between the probe vs. beads; (2) incubation of the probe with beads first vs. with lysates first; (3) the incubation time of the probe–bead complexes with lysates. As shown in Figure 1E, incubating the probe with beads and then with lysates yielded stronger bands than incubating the probe with lysates first, especially when the ratio of probe vs. the binding affinity of the beads for the single-strand oligonucleotide was higher than 1:4, suggesting that excess probes prevented probe/IRP complexes from binding to the beads. Secondly, the band intensities increased with the decrease in probe/bead ratio and reached a plateau when the ratio was 1:4 or lower, even when the probe was first incubated with the beads, suggesting that a high density of probes bound to the beads may encroach on the space required by the full probe/IRP complex and prevent IRPs from binding to the beads. The above results indicated that the ratio of the probe vs. the binding affinity of the beads should be 1:4, and it is better to incubate the probe with beads first and then with lysates. Thus, in the following experiments, we used 5 pmol of the IRE probe per 100 µg beads, which theoretically could isolate as much as 500 ng of IRP proteins.

Next, we incubated the probe/bead complexes and cell lysates for various incubation times from 5, 10, 20, to 40 min and found that the band intensities of the pull-down (PD) and the flow-through (FT) fraction did not change (Figure 1F), suggesting that the IRP/probe/bead complex formed within 5 min. Of note, almost all IRP2 in the lysates was depleted by the IRE/bead complexes. To test the binding capacity of the 5 pmol IRE probe/100 µg bead complexes, we incubated the IRE/bead complexes with Hep3B cell lysates of various quantities, from 50 µg to 1000 µg. The results (Figure 1G) showed that the IRP1 and IRP2 band intensities increased with the increasing amount of input lysates in a semi-log manner up to 800 µg, suggesting that this method can measure IRP activity in a broad input-lysate range.

### 2.2. Utilizing Biotinylated IRE to Analyze IRP Activity under Variable Iron Conditions

**Figure 2:**
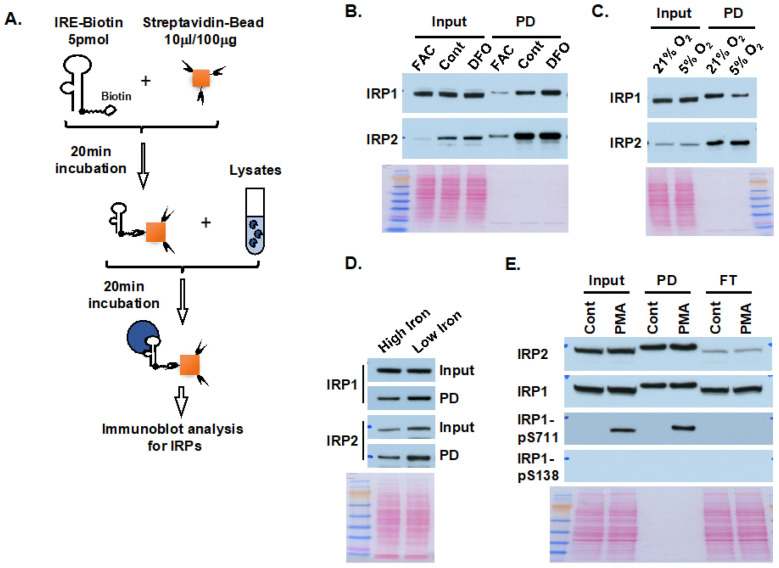
Use of biotinylated-IRE to analyze IRP activity in various iron conditions. (**A**) Optimized protocol of using biotinylated IRE probe to analyze IRP activity. (**B**) IRP activity in Hep3B cells treated with 100 μM ferric ammonium citrate (FAC), control (Cont) or deferoxamine (DFO) for 16 h. (**C**) IRP activity in Hep3B cells incubated in 21% or 5% O_2_, for 16 h. (**D**) IRP activity in kidney lysates from mice maintained on high-iron (H) or low-iron (L) diets for 3 months. (**E**) IRP activity and IRP1 phosphorylation in Hep3B cells treated with 200 nM phorbol 12-myristate 13-acetate (PMA) for 3 h. Results indicated that PMA induced IRP1-S711 phosphorylation, and phosphorylated IRP1 were present in IRE-binding form only. Ponceau S staining was used as a loading control. 25 μg lysates were loaded per lane in input, and equivalent amounts were loaded in pull-down (PD) samples. FT, flow through.

Afterward, we used the optimized protocol (Figure 2A) to measure IRP activity in cultured cells and mice under various iron conditions to test the feasibility of the method. First, Hep3B cells were treated with 100 µM ferric ammonium citrate (FAC) or deferoxamine (DFO) for 16 h to manipulate the cellular iron status. In the input fraction (Figure 2B), IRP2 levels were decreased by FAC treatment and increased by DFO treatment, whereas there were no changes in the IRP1 protein levels. In the IRE pull-down fraction, the changes in IRP2 were the same as those of the input; by contrast to the unchanged IRP1 levels in the input, the IRP1 band in the IRE pull-down fraction was decreased by FAC and increased by DFO treatment. These results are consistent with the notion that IRP2 is regulated at protein levels, whereas IRP1 converts between cytosolic aconitase and IRE-binding protein according to cellular iron and oxidation levels [1,2,3,4]. Second, Hep3B cells were maintained at 21% or 5% O_2_ for 16 h (Figure 2C). In the input fraction, there were no changes for IRP1 levels and a slight increase for IRP2 when switching from 21% to 5% O_2_ treatment; in the IRE pull-down fraction, 5% O_2_ treatment dramatically decreased the IRE-binding activity of IRP1. The results are consistent with the notion that hypoxia stabilizes the iron–sulfur cluster to decrease the IRP1-IRE-binding activity, and thereby IRP2 becomes dominant at physiological levels of O_2_ [15]. Third, we measured IRP activity in the kidney lysates of mice maintained on high-iron or low-iron diets for 3 months. As shown in Figure 2D, IRP2 levels increased in both input and pull-down samples of mice on the low-iron diet; by contrast, IRP1 levels did not change in the input samples but dramatically increased in the PD sample of mice on the low-iron diet. The above results confirmed that this method could measure IRP activity in both cultured cells and animal tissues under various iron conditions.

In vitro experiments with oligopeptides have previously suggested that Ser-138 and Ser-711 of IRP1 are potential phosphorylation sites upon protein kinase C (PKC) activation by phorbol 12-myristate 13-acetate (PMA) treatment, and the phosphorylation might promote IRE-binding activity [16]. However, in HEK293 cells treated with PMA, Ser-711, but not Ser-138, was phosphorylated, and the IRP1 bearing a phosphomimetic S711E substitution did not have IRE-binding activity, suggesting that the role of IRP1 phosphorylation at Ser-711 remains to be determined [17]. Because this method separates the IRE-binding forms from the rest of the IRP proteins, we measured the phosphorylation of IRP1 in these two fractions in lysates of Hep3B cells treated with 200 nM PMA for 3 h. Results (Figure 2E) showed that PMA treatment did not dramatically change the IRP1 and IRP2 expression levels or IRE-binding activity, but it induced the phosphorylation at Ser-711 but not at the Ser-138 site, which is consistent with the HEK293 cells [17]. Notably, pSer711-IRP1 only presented in the IRE-binding fraction, and there was no pSer711-IRP1 in the flow-through, indicating that phosphorylated IRP1 lost the capacity to coordinate an FeS cluster and/or only Ser711 in apo-IRP1 could be accessed and phosphorylated by PKC.

### 2.3. Biotinylated-IRE Probe Revealed a Truncated IRE-Binding IRP2 Isoform

**Figure 3:**
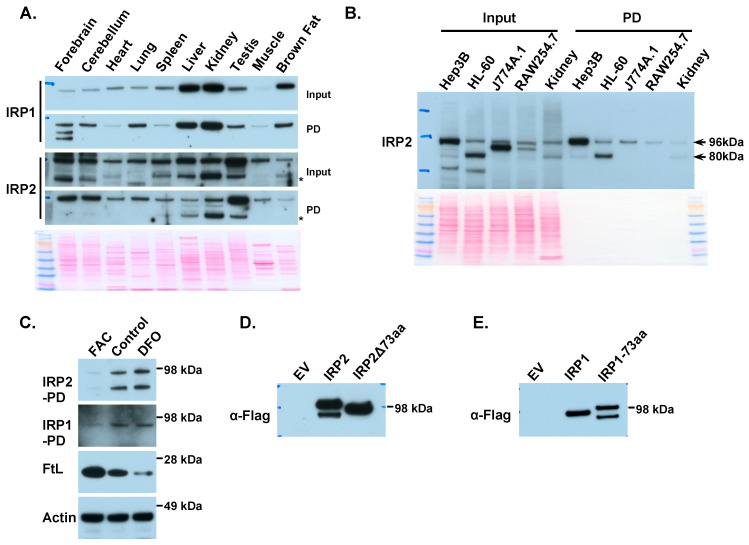
Biotinylated IRE probe revealed a truncated IRP2 isoform. (**A**) IRP activity in different mouse tissues. IRP1 showed high activity in the kidneys, liver, and brown fat, while IRP2 displayed high activity in the forebrain, cerebellum, and testis. Of note, below the expected IRP2 band, there is another band (indicated by *) at around 80 kDa in tissues including the kidneys, liver, and testis. Since this band is also present in IRE pull-down fractions, indicating that this truncated isoform keeps the IRE-binding activity. (**B**) Analysis of IRP2 activity in cell lines revealed that the truncated IRP2 isoform is abundant in HL-60 cells. Notedly, in the macrophage cell lines J774A.1 and RAW254.7, there is a strong band at around 90 kDa below the 96 kDa IRP2 bands; however, the 90 kDa band does not have IRE-binding activity. (**C**) The 80 kDa truncated IRP2 isoform displayed a similar trend as the intact IRP2 isoform in HL-60 cells treated with 100 μM ferric ammonium citrate (FAC) or deferoxamine (DFO) for 16 h. IRP1 and IRP2 immunoblots for IRE-binding fraction; immunoblots for ferritin-L and actin in cell lysates. (**D**) The truncated IRP2 product was generated from a site in the 73aa insert. HL-60 cells were transfected with empty vector, pCMV6-IRP2-f/m or pCMV6-IRP2∆73aa-f/m, then 25 μg lysates were used for the IRP activity assay and immunoblotted with anti-Flag antibody. The truncated IRP2 isoform disappeared after the 73aa insert was deleted in IRP2. (**E**) Insertion of the 73aa IRP2 insert into IRP1 generated a truncated IRP1 isoform. HL-60 cells were transfected with empty vector, pCMV6-IRP1-f/m or pCMV6-IRP1+73aa-f/m, then 25 μg lysates were used for the IRP activity assay and immunoblotted with anti-Flag antibody.

Next, we analyzed the expression and activity of IRP1 and IRP2 in various mouse tissues (Figure 3A). IRP1 showed high expression in the kidney, liver, and brown fat, which is consistent with the previous literature [18]. Accordingly, the IRE-binding IRP1 activity was high in these three tissues. Interestingly, IRP1 activity was also notable in the lung, indicating that this tissue contains more IRE-bound IRP1, possibly due to high oxygen tension. This finding is consistent with the role of IRP1 in the pulmonary vascular system, as demonstrated in IRP1 knockout mice [19]. Compared to IRP1, IRP2 protein was more ubiquitously expressed, with high activity in the testis, forebrain, and cerebellum. Interestingly, a band at about 80 kDa was present in the kidney, liver, and testis, below the band of IRP2 at about 96 kDa. This band was also present in the pull-down fraction, indicating that it had IRE-binding activity and probably represented a truncated but active IRP2 isoform. To verify this result, we analyzed IRP2 expression and activity in a few cell lines using mouse kidney lysates as a control. The results (Figure 3B) showed that the truncated IRP2 band was also present in the human hepatocellular carcinoma cell line Hep3B and the human promyelocytic cell line HL-60. Notably, other bands in the IRP2 immunoblots (Figure 3A,B) were not present in the IRE-binding fraction, suggesting that those bands were either not IRP2 or were not active IRP2. Additionally, a band slightly below 96 kDa was present in the macrophage cell lines J774A.1 and RAW264.7 and not present in IRE-binding fraction but could also be recognized by IRP2 antibody for other epitopes, indicating that the band may be an inactive IRP2.

To investigate the function of the truncated 80 kDa IRP2 isoform, we analyzed its expression under different iron conditions in HL-60 cells treated with 100 μM of FAC or 100 μM of DFO for 16 h. The intracellular iron statuses were verified by decreased ferritin expression and increased IRP1 activity from FAC to DFO treatments (Figure 3C). Accordingly, the full-length IRP2 band dramatically increased with the decrease in iron availability, and the truncated IRP2 band demonstrated a similar trend as the intact IRP2, indicating that it probably originated from the full-length IRP2. Though IRP1 and IRP2 share 60% of sequence homology, IRP2 has a unique 73 amino acid insert spanning from amino acids 139 to 211. Since IRP1 does not have a similar truncated band and there is a size difference of about 16 kDa between the intact IRP2 band (~96 kDa) and the truncated band (~80 kDa), we envisaged that the truncated IRP2 might be a proteolytic product cleaved from a site inside the 73 amino acid insert. Thus, we overexpressed a C-terminal Flag-tagged full-length IRP2 (IRP2-f/m) or IRP2 lacking the 73 amino acid insert (IRP2Δ73aa-f/m) in HL-60 cells. Immunoblot results (Figure 3D) showed that the full-length IRP2 plasmid yielded a band at the expected size and a band below, whereas the IRP2Δ73aa plasmid only generated one band. We also inserted the 73 amino acid human IRP2 sequence into IRP1, and immunoblots (Figure 3E) showed that IRP1-73aa plasmids generated a full-length band and a band below, while the IRP1 plasmids only produced one band. The above results indicate that the truncated IRP2 is a product generated by proteolytic cleavage of IRP2 at a site inside the 73 amino acid insert.

### 2.4. Dodecyl Sulfate Levels in the Loading Buffer and Denature Temperature on IRP Band Intensity

**Figure 4:**
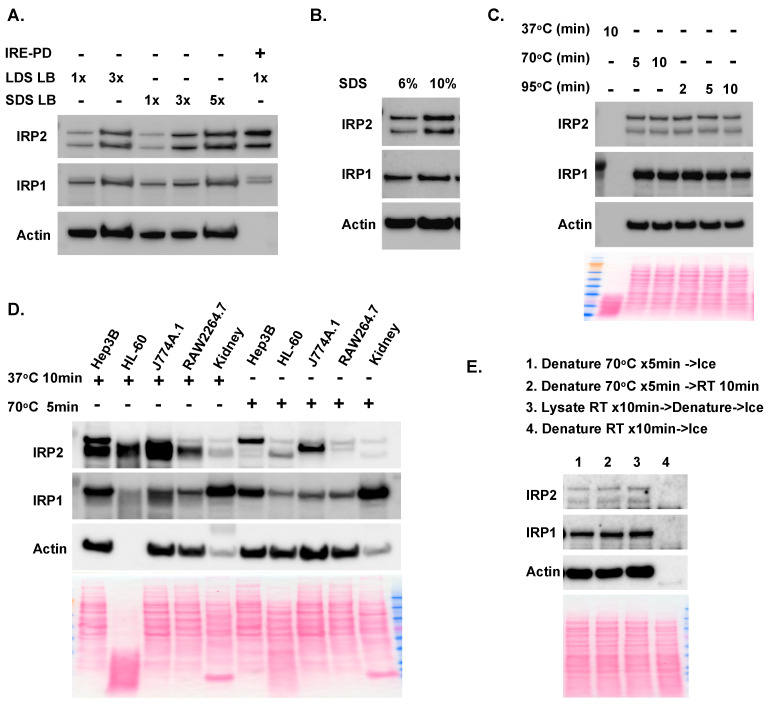
The effect of dodecyl sulfate levels and denaturation temperature on Western blot analysis of IRPs. (**A**) IRP immunoblot of HL-60 cell lysates with different concentration of lithium dodecyl sulfate (LDS) or sodium dodecyl sulfate (SDS) loading buffers. (**B**) IRP immunoblot of HL-60 cell lysates with 6% or 10% SDS in the loading buffer. The samples in A. and B. were denatured at 70 °C for 5 min. (**C**) IRP immunoblot of HL-60 cell lysates denatured at 37 °C, or 70 °C, or 95 °C for various time with 10% SDS loading buffer. Results indicated that proteins were degraded when denaturing at 37 °C for 10 min. (**D**) IRP immunoblot of different cell lysates denatured at 37 °C for 10 min or 70 °C for 5 min in 10% SDS loading buffer. Results indicated that denature at 37 °C for 10 min induced various levels of IRP degradation in different cell lysates. (**E**) IRP immunoblot of HL-60 cell lysates denatured with different conditions in 1x LDS loading buffer. Results indicated that addition of dodecyl sulfate activated protein degradation in room temperature.

While running input and IRE-PD samples on the same gel, we observed an interesting phenomenon: sometimes, the IRP2 band intensity in the IRE-PD samples was stronger than that of the input, even though IRP proteins were from equivalent quantities of cell lysates (Figure 1D–F). We reasoned that this might be due to a higher loading buffer-to-proteins ratio in the IRE-PD samples than in the input, as there were significantly fewer proteins after IRPs were purified from cell lysates. Thus, we compared the effects of different concentrations of loading buffers on IRP band intensity in HL-60 cell lysates. Results (Figure 4A) showed that 3x LDS loading buffer dramatically increased the IRP1 and IRP2 band intensity, and SDS loading buffer also increased IRP band intensity in a concentration-dependent manner. Since one major role of the loading buffer is to denature proteins with dodecyl sulfate, and there were 5% and 8.3% SDS in 3x and 5x SDS loading buffer, respectively, we tested the effect of 6% and 10% SDS in 1x loading buffer. As expected, 10% SDS dramatically increased the IRP2 band intensity (Figure 4B), indicating that 1–2% of dodecyl sulfate in the regular loading buffer might not be enough to denature IRP proteins.

Given that the primary role of dodecyl sulfate in the loading buffer is to denature proteins, we then tested the impact of different denaturing temperatures on IRP band intensity in the 10% SDS loading buffer with HL-60 cell lysates. Results (Figure 4C) showed that the band intensities were similar in samples denatured at either 70 °C for 5 or 10 min or 95 °C for 2 or 5 min, though the band intensity was slightly weaker after denaturing at 95 °C for 10 min. Surprisingly, no bands were detected in samples denatured at 37 °C for 10 min, and Ponceau S staining showed that the proteins were completely degraded. Denaturing at 37 °C or room temperature is a common practice for membrane protein immunoblots, as high temperatures likely cause membrane protein aggregation and prevent the proteins from entering the gel [20]. We tested denaturing at 37 °C for 5 min in 10% SDS loading buffer for other cells, and results (Figure 4D) showed that IRPs, especially IRP2, were more or less degraded in Hep3B, J774A.1, RAW264.7, and kidney lysates, as indicated by the smear under the IRP bands.

To understand at which step the proteins are degraded, we tested different denaturing procedures: (1) mixing HL-60 lysates with 1x LDS loading buffer, denaturing at 70 °C for 5 min, then keeping on ice; (2) mixing HL-60 lysates with 1x LDS loading buffer, denaturing at 70 °C for 5 min, then keeping at room temperature; (3) incubating HL-60 lysates at room temperature for 10 min, then mixing with 1x LDS loading buffer, denaturing at 70 °C for 5 min, then keeping on ice; and (4) mixing HL-60 lysates with 1x LDS loading buffer, denaturing at room temperature for 10 min, then keeping on ice. Results (Figure 4E) showed that the IRP band intensities were similar in protocols 1–3, but the bands disappeared after mixing with loading buffer and incubating at room temperature for 10 min. These results indicated that the addition of loading buffer activated some proteases that degrade proteins at 37 °C or room temperature, and temperatures of 70 °C and higher could inactivate the proteases and prevent protein degradation.

## 3. Discussion

The IRE-binding activity of IRPs changes according to cellular iron levels and has been used as an indicator of the cellular labile iron pool. In contrast to the calcein-AM-based assay, which measures redox-active iron within live cells based on fluorescence difference with and without iron chelators [21,22], the IRE-binding assay measures IRP activity in cell and tissue lysates, offering insights into the iron status and regulatory mechanisms that control cellular iron homeostasis. Biotinylated-IRE probes had been used initially for affinity purification of IRPs [9], and recently they were also used in a few papers to measure the IRE-binding activity [10,11,12,13]; however, the method has not led to widespread use as an assay to measure IRP activity due to a lack of technical details. Here, we exploited and optimized the method and revealed that 5 pmol probes/100 µg M280 bead complexes, specifically, probes/beads in a ratio of 1:4, could measure IRP activity in various iron conditions (Figure 3). Compared to the traditional EMSA method with the ^32^p-IRE probe, this method does not use a radioisotope, and the biotinylated IRE can be stored for years, making it more practical and cost-effective. In addition, because the method separates IRE-binding proteins from total IRP proteins, it can reveal some information that the EMSA method does not. With this method, we showed that IRP1 with Ser-711 phosphorylation was present in IRE-binding form only in PMA-treated Hep3B cells, indicating that the phosphorylation on Ser-711 potentially promotes IRE-binding activity [16].

Antibody specificity is a common issue for immunoblots, and many researchers have encountered the problem of IRP2 band specificity, even in tissues that are rich in IRP2. This method, which uses the biotinylated IRE to separate IRE-binding proteins from other proteins in lysates, increases the specificity of IRP2 antibodies and facilitates research on IRP2. Using this method, we discovered an 80 kDa truncated IRP2 isoform that is generated by cleavage of IRP2 at a site within the 73aa IRP2 insert (Figure 3D,E). The truncated IRP2 showed a similar trend as the full-length IRP2 in response to FAC and DFO treatment. The appearance of the 80 kDa truncated band was not affected by the serine proteinase inhibitor phenylmethylsulfonyl fluoride (PMSF), indicating that the truncation form might exist endogenously. A truncated IRP2 band was previously reported in rat liver lysates with an N-terminal sequence starting from amino acid 195 of rat IRP2 [23]. An 80 kDa truncated IRP2 band resulting from proteolytic cleavage of IRP2 was also reported in H1299 lung cancer cells with cleavage sites between amino acids 159–166 [24]. Our results, combined with those from two previous studies, confirm that the 73aa insert is susceptible to proteolytic cleavage. We also conducted a comparison of the affinity of the truncated IRP2 form and full-length IRP2 with the H-ferritin IRE. Additionally, we examined the stability of these two forms in HL-60 cells but did not find a significant difference. Overexpressed IRP2 lacking the N-terminal 166 aa did not exhibit IRE-binding activity, which suggests that the 80 kDa truncated IRP2 arises from the proteolytic cleavage of mature IRP2 protein. Beneath the full-length IRP2 band, a robust band is observed in the macrophage cell lines J774A.1 and RAW254.7. This band lacks IRE-binding activity but can be recognized by another IRP2 antibody targeting a different epitope. This implies that the band represents an inactive IRP2 and likely originates from a modification specific to macrophages.

Regular denaturing loading buffers typically have a final concentration of 1–2% dodecyl sulfate. In investigating why IRP2 bands were stronger in IRE-PD samples compared to equivalent amounts of the input, we discovered that dodecyl sulfate significantly increased IRP band intensity in a concentration-dependent manner (Figure 4). The basis for this observation is not entirely clear; one possibility is that a high concentration of dodecyl sulfate may relax the protein structure, enabling the antibody to better access the epitope. Nevertheless, it might be worth using an increased concentration of SDS when encountering issues in immunoblot analysis of IRP proteins or possibly other proteins as well.

When using immunoblotting to analyze membrane proteins, including transferrin receptor 1, ferroportin, and divalent metal transporter 1, the protein samples must be denatured at 37 °C or room temperature, as high temperatures can lead to the aggregation of these membrane proteins, compromising immunoblot analysis [20]. However, our results showed that the addition of loading buffer activated some proteases that degrade proteins at 37 °C or room temperature, especially in HL-60 cells. This suggests that, for the immunoblot analysis of membrane proteins, the optimal protocol might involve directly loading samples onto the gel for analysis after mixing with loading buffer.

In summary, the biotinylated-IRE method is more practical and cost-effective, by isolating IRE-binding forms from other proteins, it can reveal information that the traditional EMSA method cannot show. Because this method can analyze IRPs isolated from mg or more input lysates, while regular immunoblotting only analyzes 15–30 μg of total proteins per lane, it is much more sensitive than regular immunoblotting. We believe that the application of this method will promote iron metabolism research.

## 4. Materials and Methods

### 4.1. Cells

Hep3B cells (MilliporeSigma, Rockville, MD, USA) were cultured in Eagle’s Minimum Essential Medium supplemented with 10% fetal bovine serum and L-glutamine. HL-60 cells were cultured in Iscove’s Modified Dulbecco’s Medium supplemented with 20% fetal bovine serum and L-glutamine. J774A.1 and RAW254.7 cell lines were cultured in RPMI-1640 medium with 10% fetal bovine serum and L-glutamine. All of these cells were cultured in 5% CO_2_ at 37 °C. For hypoxia treatment, Hep3B cells were incubated with 5% O_2_ and 5% CO_2_ for 16 h. The cells were treated with 100 μM of ferric ammonium citrate (FAC) or deferoxamine (DFO) for 16 h to deactivate or activate IRP proteins. For phorbol 12-myristate 13-acetate (PMA) treatment, the cells were incubated with 200 nM of PMA for 3 h.

### 4.2. Plasmids and Transfection

pCMV6-IRP2-f/m vector was created by cloning human IRP2 into pCMV6-entry f/m vector with SgfI and MluI sites, which expresses IRP2 with a C-terminal Flag and Myc tags. pCMV6-IRP2Δ73aa-f/m vector was created by deleting the 73aa insert sequence (residues 139–211) from IRP2 of pCMV6-IRP2-f/m vector with QuickChange Site-Directed Mutagenesis Kit (Agilent, Santa Clara, CA, USA) with the following primers: sense, 5′-tttacaaattgacttcagtaaatgtgcagaaacagtgttaaaaaatcaagaagtag-3′; antisense, 5′-ctacttcttgattttttaacactgtttctgcacatttactgaagtcaatttgtaaa-3′. pCMV6-IRP1-f/m vector was created by cloning human IRP1 into pCMV6-entry f/m vector with HindIII and NotI sites. To create the pCMV6-IRP1+73aa-f/m vector, we initially cloned the IRP2 73aa (residues 139–211) into human IRP1 via PCR then inserted the product into pCMV6-entry f/m vector with HindIII and NotI sites. The plasmids were transfected into HL-60 cells with the Neon^TM^ transfection system according to the manufacturer’s protocol, and the cell lysates were prepared 7 h after transfection.

### 4.3. Mice

All the mouse experiments were carried out following protocols approved by the Animal Care and Use Committee of NICHD and met NIH guidelines for the humane care of animals. The IRP1^−/−^ and IRP2^−/−^ mice were described previously [19,25]. The mice were maintained on a normal diet (350 mg iron/kg). For high-iron or low-iron treatment, the mice were maintained on a high-iron diet (1.6 g iron/kg) or low-iron diet (2–6 mg iron/kg) for at least three months. All diets were manufactured by Envigo (Formerly Harlad Teklad, Indianapolis, IN, USA).

### 4.4. Protocol

The H-ferritin IRE was used as the IRE probe, and the sequence is as follows: 5′-GGAGUUCCUGCUUCAACAGUGCUUGGACGGAACUCC-3′; the sequence of the mutated IRE probe is 5′-GGAGUUCCUGCUUCAAAGUGCUUGGACGGAACUCC-3′. The probes were ordered from Eurofins Scientific with 3′-biotinylation. The probes were resuspended with water to 10 µM (10 pmol per microliter) solution and then aliquoted and kept at −80 °C. Prior to performing the assay, the probes were denatured at 95 °C for 1 min and then cooled down to room temperature for 5 min to anneal to the stem-loop structure before being kept on ice for the IRE-binding activity assay.

For preparing cell lysates, Hep3B cells cultured in 10 cm plates were washed once with ice-cold PBS and then were lifted with a cell lifter to 5 mL of cold PBS. The cells were centrifuged at 500× *g* for 5 min, then the pellets were resuspended in 5 volumes of lysis buffer (40 mM KC1, 25 mM Tris-C1, pH 7.5, 1% Triton X-100, 1x Halt™ Protease Inhibitor Cocktail (ThermoFisher Scientific, Waltham, MA, USA), 1 mM DTT, and 20 u/mL RNase Inhibitor (ThermoFisher Scientific)) and kept on ice for 20 min. Then, the lysates were centrifuged at 20,000× *g* for 5 min at 4 °C, and the supernatant was isolated, aliquoted, and kept at −80 °C for the IRE-binding activity assay. For preparing tissue lysates, mouse tissues were sliced into small pieces on ice then were homogenized with 5 volumes of lysis buffer with Bullet Blender tissue homogenizer (Next Adavance, Inc., Troy, NY, USA), followed by centrifugation at 20,000× *g* for 5 min at 4 °C to isolate the supernatant. The protein concentration was determined with the Bradford method (Bio-Rad, Hercules, CA, USA).

DynaBead M-280 Streptavidin was ordered from ThermoFisher, and 100 micrograms (10 μL) of beads were used per sample except when otherwise specified. The required quantity of DynaBeads was washed twice with Buffer I (DEPC-treated 0.1 M NaOH, 0.05 M NaCl) and once with Buffer II (DEPC-treated 0.1 M NaCl) before being washed twice with lysis buffer (40 mM KC1, 25 mM Tris-C1, pH 7.5, 1% Triton X-100). For the optimal protocol, 100 µg of beads were incubated with 5 pmol of biotinylated IRE in 200 µL of lysate buffer for 20 min at room temperature in a shaker and then washed twice with lysate buffer. The bead/probe complexes were incubated with 100 µg lysates in a final volume of 200 µL at room temperature on a shaker for 20 min. At the end of the incubation, the bead/probe/IRP complexes were washed three times with lysate buffer, spun down at 1000× *g* for 1 min, and the residual buffer was removed with a magnet and aspirator then mixed with 100 μL of 1x NuPAGE™ LDS Sample Buffer (ThermoFisher Scientific) with 50 mM DTT. For the input, 100 µg of lysates were mixed with LDS Sample Buffer unless otherwise specified to a final volume of 100 µL. The samples were incubated at 85 °C for 5 min unless otherwise specified to denature the proteins and then were loaded to 25 µL (equivalent to 25 μg) each to 4–12% Bis-Tris gels (ThermoFisher Scientific) and separated at 200 V for 45 min with NuPAGE™ MES buffer (ThermoFisher Scientific). After being transferred to the nitrocellulose membrane, the blots were blocked for 30 min with blocking buffer (5% non-fat milk, 0.1% Tween-20 in PBS, pH7.4) and then incubated overnight with rabbit-anti-IRP1 or IRP2 antibodies. The blots were incubated with HRP-anti-rabbit antibody for 1 h, washed with PBST (0.1% Tween-10, 1x PBS, pH7.4) 3 times, and then exposed to x-film or Chemidoc Imager after incubation with SuperSignal West Pico Substrates (ThermoFisher Scientific). The rabbit-anti-IRP1 was prepared against the N-terminal 16aa of mouse IRP1, the rabbit-anti-IRP2 antibody was prepared against aa 137–209 of human IRP2, and the IRP1-pS138 (ab63260) and IRP1-pS711 (ab62451) antibodies were purchased from Abcam (Waltham, MA, USA).

For testing the IRP band intensity in different loading buffers and denaturation temperatures (Figure 4), cell lysates were mixed with 4x NuPAGE™ LDS Sample Buffer or 6x SDS loading buffer (280 mM Tris-Cl pH6.8, 30% glycerol, 6. mM EDTA, 10% SDS, 882 mM beta-mercaptoethanol, 0.04% bromophenol blue) in different ratios to obtain the final concentration of loading buffer of 1x, 3x, or 5x as required. The 6% and 10% SDS loading buffers were prepared by adjusting SDS levels to obtain the final concentration of 6% or 10% SDS, while the remaining gradients were the same as the 1x SDS loading buffer.

### 4.5. Statistical Analyses

The regression in Figure 1G was performed with GraphPad Prism 10. Each of the immunoblot results was repeated at least two times.

## Data Availability

All data in the paper are available for sharing.
